# Supplementary material for: Multi‐dataset identification of innovative feature genes and molecular mechanisms in keratoconus
Source: J Cell Mol Med. 2024 Sep 19;28(18):e70079. doi: 10.1111/jcmm.70079 (PMC11412914; doi:10.1111/jcmm.70079)
Supplement: Supplementary file 2 — Material S2. [file JCMM-28-e70079-s002.zip › 7.GSEA analysis/6.SNORA74B/KEGG_PROSTATE_CANCER.html]

Details for gene set KEGG\_PROSTATE\_CANCER[GSEA]

|  || Dataset | SNORA74B.group.cls#h\_versus\_l |
| Phenotype | group.cls#h\_versus\_l |
| Upregulated in class | l |
| GeneSet | KEGG\_PROSTATE\_CANCER |
| Enrichment Score (ES) | -0.401648 |
| Normalized Enrichment Score (NES) | -1.6946325 |
| Nominal p-value | 0.0019569471 |
| FDR q-value | 0.24581702 |
| FWER p-Value | 0.404 |
Table: GSEA Results Summary

  

Fig 1: Enrichment plot: KEGG\_PROSTATE\_CANCER      
 Profile of the Running ES Score & Positions of GeneSet Members on the Rank Ordered List

  

| SYMBOL | TITLE | RANK IN GENE LIST | RANK METRIC SCORE | RUNNING ES | CORE ENRICHMENT || 1 | PIK3R5 | na | 170 | 0.800 | 0.0544 | No |
| 2 | INSRR | na | 1547 | 0.412 | 0.0453 | No |
| 3 | PDGFRA | na | 4146 | 0.255 | -0.0108 | No |
| 4 | SOS1 | na | 5971 | 0.193 | -0.0492 | No |
| 5 | IGF1R | na | 6399 | 0.180 | -0.0482 | No |
| 6 | CCNE2 | na | 7159 | 0.158 | -0.0584 | No |
| 7 | KLK3 | na | 7466 | 0.149 | -0.0561 | No |
| 8 | E2F2 | na | 7913 | 0.138 | -0.0588 | No |
| 9 | BRAF | na | 8089 | 0.134 | -0.0539 | No |
| 10 | PIK3R1 | na | 8116 | 0.133 | -0.0448 | No |
| 11 | IGF1 | na | 8530 | 0.124 | -0.0475 | No |
| 12 | IKBKB | na | 8763 | 0.119 | -0.0454 | No |
| 13 | MTOR | na | 8938 | 0.115 | -0.0419 | No |
| 14 | CCNE1 | na | 8992 | 0.114 | -0.0350 | No |
| 15 | AKT2 | na | 9380 | 0.105 | -0.0384 | No |
| 16 | KRAS | na | 9766 | 0.097 | -0.0423 | No |
| 17 | PDGFA | na | 9861 | 0.095 | -0.0379 | No |
| 18 | AR | na | 10468 | 0.084 | -0.0492 | No |
| 19 | CREBBP | na | 10525 | 0.083 | -0.0447 | No |
| 20 | MDM2 | na | 11211 | 0.071 | -0.0592 | No |
| 21 | PDPK1 | na | 11353 | 0.069 | -0.0581 | No |
| 22 | TCF7L2 | na | 12663 | 0.051 | -0.0921 | No |
| 23 | PTEN | na | 12756 | 0.050 | -0.0911 | No |
| 24 | ERBB2 | na | 12767 | 0.050 | -0.0877 | No |
| 25 | HSP90AA1 | na | 12770 | 0.050 | -0.0840 | No |
| 26 | PIK3R2 | na | 12946 | 0.047 | -0.0856 | No |
| 27 | PIK3CA | na | 13079 | 0.046 | -0.0859 | No |
| 28 | RB1 | na | 13606 | 0.040 | -0.0981 | No |
| 29 | SOS2 | na | 13685 | 0.039 | -0.0975 | No |
| 30 | PDGFRB | na | 14565 | 0.029 | -0.1207 | No |
| 31 | MAP2K2 | na | 14904 | 0.025 | -0.1285 | No |
| 32 | CCND1 | na | 15223 | 0.022 | -0.1361 | No |
| 33 | CREB3 | na | 15657 | 0.018 | -0.1472 | No |
| 34 | PIK3CB | na | 15769 | 0.017 | -0.1492 | No |
| 35 | CREB3L1 | na | 15845 | 0.016 | -0.1501 | No |
| 36 | CHUK | na | 16402 | 0.010 | -0.1654 | No |
| 37 | INS | na | 16698 | 0.007 | -0.1734 | No |
| 38 | MAPK3 | na | 16753 | 0.007 | -0.1744 | No |
| 39 | E2F3 | na | 17092 | 0.004 | -0.1839 | No |
| 40 | EGF | na | 17351 | 0.001 | -0.1913 | No |
| 41 | GSTP1 | na | 17706 | -0.003 | -0.2013 | No |
| 42 | CTNNB1 | na | 17783 | -0.003 | -0.2032 | No |
| 43 | GSK3B | na | 17871 | -0.004 | -0.2054 | No |
| 44 | AKT1 | na | 18366 | -0.010 | -0.2189 | No |
| 45 | CREB3L3 | na | 18368 | -0.010 | -0.2182 | No |
| 46 | SRD5A2 | na | 18577 | -0.013 | -0.2232 | No |
| 47 | ATF4 | na | 18691 | -0.014 | -0.2255 | No |
| 48 | PIK3CG | na | 18884 | -0.016 | -0.2298 | No |
| 49 | FOXO1 | na | 18961 | -0.017 | -0.2308 | No |
| 50 | BAD | na | 19265 | -0.021 | -0.2380 | No |
| 51 | TP53 | na | 19572 | -0.024 | -0.2451 | No |
| 52 | EGFR | na | 19596 | -0.024 | -0.2440 | No |
| 53 | HRAS | na | 19699 | -0.025 | -0.2451 | No |
| 54 | HSP90AB1 | na | 19743 | -0.026 | -0.2444 | No |
| 55 | CDKN1B | na | 20194 | -0.030 | -0.2551 | No |
| 56 | PIK3CD | na | 20354 | -0.032 | -0.2573 | No |
| 57 | MAPK1 | na | 20606 | -0.035 | -0.2620 | No |
| 58 | IKBKG | na | 20820 | -0.038 | -0.2653 | No |
| 59 | TGFA | na | 20989 | -0.040 | -0.2672 | No |
| 60 | RAF1 | na | 21074 | -0.041 | -0.2666 | No |
| 61 | CREB3L4 | na | 21237 | -0.043 | -0.2681 | No |
| 62 | CASP9 | na | 21843 | -0.050 | -0.2819 | No |
| 63 | CREB3L2 | na | 22159 | -0.054 | -0.2870 | No |
| 64 | FGFR2 | na | 24244 | -0.085 | -0.3409 | No |
| 65 | HSP90B1 | na | 24525 | -0.090 | -0.3423 | No |
| 66 | GRB2 | na | 24997 | -0.098 | -0.3486 | No |
| 67 | EP300 | na | 25385 | -0.105 | -0.3520 | No |
| 68 | CREB1 | na | 25963 | -0.116 | -0.3600 | No |
| 69 | NFKB1 | na | 26534 | -0.128 | -0.3670 | No |
| 70 | CDK2 | na | 27098 | -0.141 | -0.3727 | Yes |
| 71 | TCF7L1 | na | 27185 | -0.144 | -0.3646 | Yes |
| 72 | E2F1 | na | 27392 | -0.149 | -0.3595 | Yes |
| 73 | ARAF | na | 27916 | -0.162 | -0.3625 | Yes |
| 74 | NRAS | na | 27969 | -0.164 | -0.3519 | Yes |
| 75 | NFKBIA | na | 28570 | -0.182 | -0.3557 | Yes |
| 76 | TCF7 | na | 28810 | -0.189 | -0.3486 | Yes |
| 77 | PIK3R3 | na | 29453 | -0.208 | -0.3517 | Yes |
| 78 | RELA | na | 29651 | -0.214 | -0.3415 | Yes |
| 79 | PDGFB | na | 30041 | -0.227 | -0.3359 | Yes |
| 80 | MAP2K1 | na | 31129 | -0.275 | -0.3469 | Yes |
| 81 | CDKN1A | na | 31225 | -0.280 | -0.3289 | Yes |
| 82 | BCL2 | na | 33746 | -0.485 | -0.3657 | Yes |
| 83 | LEF1 | na | 34351 | -0.633 | -0.3362 | Yes |
| 84 | PDGFC | na | 34382 | -0.645 | -0.2892 | Yes |
| 85 | NKX3-1 | na | 34408 | -0.652 | -0.2416 | Yes |
| 86 | PDGFD | na | 34541 | -0.723 | -0.1918 | Yes |
| 87 | CREB5 | na | 34618 | -0.816 | -0.1335 | Yes |
| 88 | AKT3 | na | 34657 | -0.880 | -0.0694 | Yes |
| 89 | FGFR1 | na | 34687 | -0.963 | 0.0012 | Yes |
Table: GSEA details [plain text format]

  

Fig 2: KEGG\_PROSTATE\_CANCER      
 Blue-Pink O' Gram in the Space of the Analyzed GeneSet

  

Fig 3: KEGG\_PROSTATE\_CANCER: Random ES distribution      
 Gene set null distribution of ES for **KEGG\_PROSTATE\_CANCER**
